# Composition and Properties of Saliva in Patients with Osteoporosis Taking Antiresorptive Drugs

**DOI:** 10.3390/ijerph20054294

**Published:** 2023-02-28

**Authors:** Hanna Sobczak-Jaskow, Barbara Kochańska, Barbara Drogoszewska

**Affiliations:** 1 Department of Maxillofacial Surgery, Medical University of Gdansk, 80-210 Gdansk, Poland; 2 Department of Conservative Dentistry, Medical University of Gdansk, 80-210 Gdansk, Poland

**Keywords:** saliva, osteoporosis, antiresorptive drugs, bisphosphonates, denosumab, oral health, systemic diseases

## Abstract

The aim of this study was to examine how the composition and properties of saliva change in people with osteoporosis who have received antiresorptive (AR) treatment, compared to patients with osteoporosis who have not yet received this treatment. Methods: The study population consisted of 38 patients with osteoporosis using AR drugs (Group I) and 16 patients with osteoporosis who had never used AR drugs (Group II). The control group consisted of 32 people without osteoporosis. Laboratory tests included determination of pH and concentrations of Ca, PO_4_, total protein, lactoferrin, lysozyme, sIgA, IgA, cortisol, neopterin, activity of amylase at rest, and stimulated saliva. The buffering capacity of stimulated saliva was also determined. Results: There were no statistically significant differences between the saliva of Group I and Group II. No statistically significant correlation was found between the amount of time using AR therapy (Group I) and the tested parameters of the saliva. Significant differences were found between Group I and the control group. The concentrations of PO_4_, lysozyme, and cortisol were higher, while concentrations of Ca ions, sIgA, and neopterin were lower, in comparison to the control group. The significant differences between Group II and the control group were smaller, and they concerned only the concentrations of lysozyme, cortisol, and neopterin. Conclusions: The saliva of people with osteoporosis subjected to AR therapy and those not subjected to AR therapy did not show statistically significant differences in terms of the examined parameters of the saliva. However, the saliva of patients with osteoporosis taking and not taking AR drugs was significantly different compared to the saliva of the control group.

## 1. Introduction

Osteoporosis is a disease of the skeletal system characterized by increased susceptibility of bones to fractures, which occur as a result of bone tissue structure disorders and low bone mass [1,2,3,4,5]. In the course of osteoporosis, there is loss of bone mass of the entire skeleton; this relationship also applies to the maxilla and the mandible [5,6,7]. Patients suffering from osteoporosis and osteopenia are exposed not only to the loss of mineral density of the jaw bones, but also to tooth loss and periodontal disease [5,7,8,9].

Due to demographic changes, osteoporosis is a growing epidemiological problem [1,2,4,10,11,12,13]. According to the data from the literature, it is estimated that over 22 million women and 5.5 million men in Europe suffer from osteoporosis [1,2]. The incidence of osteoporosis increases with age and is more common in women [1,4,5,11].

Due to the chronic nature of osteoporosis, it requires a long-term treatment strategy [13]. Medications used in the treatment of osteoporosis affect the basic processes during bone tissue remodeling, i.e., resorption (antiresorptive (AR) drugs) and/or bone formation (anabolic drugs) [3,13]. The major goals of treatment for osteoporosis are prevention of fractures and increase in bone mineral density (BMD) [2,12]. Bisphosphonates and denosumab are commonly prescribed treatment forms for osteoporosis [1,3,10,11,13,14].

Bisphosphonates are synthetic derivatives of pyrophosphates and have a strong AR effect [1,5,14,15,16,17]. The basic mechanism of action of bisphosphonates, i.e., inhibition of bone resorption, results from their high affinity for bone mineral components and binding to hydroxyapatite crystals [3,5,14,16,17]. Orally administered bisphosphonates are the most commonly used first-line drugs in osteoporosis [1,13,18]. This group includes alendronate, risedronate, and ibandronate—for daily, weekly, or monthly use, depending on the drug [11,13,15]. Ibandronate can also be used intravenously, with injections being given every three months.

Denosumab is a human monoclonal antibody that has a different mechanism of AR action than bisphosphonates [3,12,17,19]. It does not incorporate the bone matrix but is directed against the receptor activator of nuclear factor kappa B ligand (RANKL) [1,11,12,15,19,20]. Prevention of the RANK/RANKL association inhibits the formation, function, and survival of osteoclasts, thereby reducing bone resorption [3,19]. Denosumab is indicated mainly for postmenopausal women with osteoporosis at high risk of fracture, or for patients who have failed or are intolerant to other available osteoporosis therapies [3,12]. It has been documented in the literature that denosumab therapy is associated with a reduced risk of vertebral, non-vertebral, and proximal femoral fractures [1,11,19]. In the treatment of osteoporosis, this drug is administered via subcutaneous injections every six months [1,3,11,12,19,20].

Studies show that the use of AR drugs in the treatment of osteoporosis is associated with the risk of medication-related osteonecrosis of the jaw (MRONJ) [5,12,13,14,15,17,18,19,20,21,22]. AR drugs impair bone turnover and cause prolonged healing of the jaw bones after surgical procedures [19]. Moreover, bisphosphonates show toxic and antiangiogenic effect on the oral epithelium [5,19]. One of the factors contributing to the occurrence of this dangerous complication is the poor condition of the oral cavity, which is associated, among others, with diseases of the teeth, periodontium, and oral mucosa, as well as with poor oral hygiene [17,19,22].

An important role in maintaining oral health is that of saliva, which is the fundamental component of the oral environment and performs a number of functions that are important for health [23,24,25,26,27]. The presence of sIgA, IgA, IgM, IgG, and IgD antibodies and non-immune components in saliva enables its antimicrobial activity through specific and non-specific immunity [23,24,25,27,28,29,30]. Among the non-specific factors, lysozyme and lactoferrin, with their antibacterial activity, should be mentioned [23,25,27,28,29,30]. Thanks to its buffering capacity and the presence of PO_4_ ions and Ca ions, saliva is involved in maintaining the balance between demineralization and remineralization of hard dental tissues, supporting the repair processes in the oral cavity [23,24,26,27,29,30]. Saliva is also involved in the perception of taste, the formation of bites, and takes part in the initial digestion of starch via salivary amylase [23,24,25,26,27,28,30]. Saliva contains ingredients that reflect the condition of the whole organism [25,27,31]. These include, among others, cortisol (a marker of psychological stress) and neopterin (a non-specific indicator of cellular immune response) [25,32,33].

The aim of this study was to examine how the composition and properties of saliva, which are important for oral health, change in people with osteoporosis who have received AR treatment, compared to patients with osteoporosis who have not yet received this treatment. The obtained results were compared with the control group. The following saliva parameters were taken into account in the study: pH, buffering capacity, Ca ions, PO_4_ ions, total protein, lactoferrin, lysozyme, sIgA, IgA, cortisol, neopterin, and amylase. Knowledge about potential changes in the composition of saliva in osteoporotic patients taking AR drugs may contribute to determining the treatment needs of this group of patients in terms in MRONJ prevention.

## 2. Materials and Methods

### 2.1. Patient Population

A total of 54 patients diagnosed with osteoporosis (49 women and 5 men) aged 55–84 years (mean value = 70.5, standard deviation (SD) = 6.5, median (Me) = 70) were examined. Group I consisted of patients with osteoporosis, in whom AR therapy was implemented. AR drugs were taken by 38 patients (34 women and 4 men) aged 55–84 years (mean value = 70.6, SD = 6.8, Me = 70). Meanwhile, 26 patients were taking ibandronate sodium, 7 patients were taking alendronate sodium, 4 patients were taking denosumab, and 1 patient was taking risedronate sodium.

The majority—31 patients (81%)—were taking drugs orally, 4 patients were taking drugs subcutaneously (11%), and only 3 patients were taking drugs intravenously (8%). The average duration of therapy with AR drugs was 36.5 months (5 patients took the drugs for 2 to 6 months—an average of 4.4 months; in 17 patients, AR therapy was carried out for a period of 15 to 33 months—an average of 23.2 months; 17 patients took drugs from 36 to 120 months—an average of 56.4 months).

Group II consisted of 16 patients with osteoporosis (15 women and 1 man) aged 62–82 years (mean value = 71.1, SD = 7.0, Me = 70), who had never used AR drugs but were qualified for the implementation of this pharmacotherapy in the future.

The control group consisted of 32 people in generally good health (18 women and 14 men) aged 51–83 years (mean value= 69.3, SD = 6.9, Me = 69), with no history of osteoporosis, and who had never been treated with AR therapy.

Patients falling into one or more of the following four groups were excluded from the study: patients with systemic infection; those with active local infection within the oral cavity; patients with a disease of the salivary glands; and those with an active oncological disease. Furthermore, patients who did not comply with the rules of saliva collection or did not consent to the study were also excluded. Saliva samples contaminated with blood were disqualified from the study. Qualification of participants for the study and saliva collection was carried out in two centers: at the Maxillofacial Surgery Clinic of the Medical University Hospital in Gdansk, and at the Dental Surgery Department of the Orlowo Medical Clinic in Gdynia. All patients who participated in the study were Caucasian. Osteoporosis was diagnosed by a rheumatologist according to the current guidelines. All osteoporotic patients underwent dual X-ray absorptiometry (DXA) scans. All participants of the study received a full dental/intraoral examination. Standard dental examination included anamnesis (medical history including information on the general health, medication taken, and smoking habits). Intraoral examination included assessment of the condition of dentition, the periodontium, oral hygiene, and mucosal condition. The results obtained will be the subject of further publications. The study protocol was approved by the Ethics Committee of the Medical University of Gdansk, Poland (NKBBN/166/2016). Ethical aspects of the research were in accordance with the World Medical Association’s Declaration of Helsinki.

### 2.2. Saliva Collection

The material for the study was mixed saliva. A resting saliva sample and a stimulated saliva sample were collected from each study participant. The spitting method was applied according to the standardized guidelines. Each participant received guidelines on the method of saliva collection. All saliva samples were collected in sterile Corning-type test tubes. Saliva was collected in the morning, 2 h after the previous meal, drink, mouthwash, and tooth brushing. Study participants did not smoke or chew gum for 2 h prior to saliva collection.

Unstimulated salivary samples were obtained by expectoration in the absence of chewing movements for a period of 5 min. When collecting stimulated saliva, the actual saliva collection was preceded by chewing of a paraffin cube for one minute, and then saliva was released for 5 min. Immediately after collection, the pH level was determined in the resting saliva, and the pH level and buffer capacity were determined in the stimulated saliva.

Then, the collected samples of resting and stimulated saliva were stored at −30 °C until the start of the biochemical tests. The concentrations of PO_4_ ions (mg%) and Ca ions (mg%), total protein (mg/mL), lactoferrin (µg/mL), lysozyme (µg/mL), sIgA (µg/mL), IgA (µg/mL), cortisol (ng/mL), neopterin (nmol/mL), and amylase activity (U/mL) were determined in the collected saliva. Laboratory tests of saliva were performed in the biochemistry laboratory of the Department of Conservative Dentistry of the Medical University of Gdansk.

### 2.3. Biochemical Analysis of Saliva

The tests of the inorganic components of saliva were carried out in non-centrifuged saliva. Sigma-Aldrich pH Test Strips 4.5–10.0 were used to measure the pH of the saliva. The concentration of Ca ions was determined by the Arsenazo III method. This method uses the metallochromogen Arsenazo III, which binds Ca ions to form a colored complex, and whose absorbance can be measured at a wavelength of 650 nm. The reagent (Alpha Diagnostics) was added to the saliva sample at a ratio of 1:100 and incubated for 1 min. Absorbance was then measured at 650 nm. The calcium concentration was calculated from the standard curve. The direct method with phosphomolybdate based on the modified method of Daly and Ertingshausen was used to determine the concentration of PO_4_ ions. The reagent (Alpha Diagnostics) was added to the saliva sample at a ratio of 1:100, incubated for 6 min, and then the absorbance was measured at 340 nm. The phosphorus concentration was calculated from the standard curve. Measurements were carried out using a Sunrise f. TECAM spectrophotometer.

The tests of organic components were carried out in centrifuged saliva. The saliva was centrifuged for 10 min at 14,500 rpm using an Eppendorf Mini Spin Plus centrifuge. The total protein concentration was determined using the modified Lowry method. For protein determination by this method, the following were used: samples of spun saliva, 0.1% BSA, Folin–Ciocâlteu reagent, CTC reagent (consisting of 0.1% CuSO_4_, 0.2% potassium sodium tartrate, and 10% Na_2_SO_4_), reagent A (consisting of 5% SDS, 0.8 N NaOH, and CTC reagent at a ratio of 2:1:1). Standard albumin solution: 0.1% BSA solution was diluted so that in the next 10 tubes the protein concentration was 10–100 µg/mL. Preparation of the samples consisted of adding 500 µL of reagent A to 500 µL of the tested protein solution, which were then thoroughly mixed and left for 10 min at room temperature. With immediate vigorous stirring, 250 µL of Folin–Ciocâlteu reagent were added. After 30 min, the absorbance at 750 nm was measured.

The protein concentration in the tested samples was calculated from the calibration curve. Measurements were carried out using the HACH LANGE DR 3900 spectrophotometer.

The concentrations of lactoferrin, lysozyme, and immunoglobulin IgA were determined using the immunoenzymatic ELISA method using non-commercial tests. The immunoenzymatic ELISA method using commercial tests was used to determine the concentration of sIgA immunoglobulin (Immuniq, Immunodiagnostik), the concentration of neopterin (Demeditec), the concentration of cortisol (R&D), and the activity of amylase (IBL). The absorbances were measured at a wavelength of 450 nm. Measurements were carried out using a Sunrise f. TECAM spectrophotometer.

The CRT^®^ buffer tests by Ivoclar Vivadent were used to measure the buffering capacity of the saliva.

### 2.4. Statistical Analysis

The statistical analyses were performed by BIO-STAT Statistical Research and Analyses Studio in Gdansk, using the statistical suite STATISTICA (data analysis software system), version 12.0 (StatSoft Inc., Tulsa, OK, USA).

Statistical significance of the differences between groups was determined using the Kruskal–Wallis test. Where statistically significant differences were obtained between groups, post hoc tests were performed (Dunn’s test). The independent chi-squared test was used for qualitative variables. In order to determine the relationship, strength, and direction between the variables, correlation analysis was used by calculating the Pearson’s correlation coefficient.

In all calculations, the statistical significance level of α = 0.05 was used.

## 3. Results

There were no statistically significant differences between the examined parameters of saliva in people with osteoporosis taking AR drugs compared to people with osteoporosis who had never been subjected to AR therapy (Table 1 and Table 2). Moreover, the buffering capacity of saliva in patients treated and not treated with AR was similar (Table 3). The average duration of therapy with AR drugs was 36.5 months (SD = 24.1, range 2–120, Me = 31.5). No statistically significant correlation was found between the duration of AR therapy in patients with osteoporosis and the examined parameters of resting and stimulated saliva.

There were significant differences in some parameters of unstimulated (US) and stimulated saliva (SS) between people taking AR drugs and the control group. In the case of inorganic components, it was found that the concentration of PO_4_ ions was statistically significantly higher only in the resting saliva of patients taking AR drugs (Me = 23.39 mg%) compared to the control group (Me = 14.62 mg%) (Table 1). However, the concentration of Ca ions was lower in resting (Me = 5.12 mg%) and stimulated saliva (Me = 3.52 mg%) in people taking AR drugs than in the control group (US: Me = 5.41 mg%; SS: Me = 4.51 mg%), but this difference was statistically significant only in the case of stimulated saliva (Table 1 and Table 2).

In the case of organic components related to the non-specific immunity of saliva, a statistically significant increase in the concentration of lysozyme was found in both resting and stimulated saliva (US: Me = 0.77 μg/mL; SS: Me = 0.64 μg/mL) in people taking AR compared to the control group (US: Me = 0.30 μg/mL; SS: Me = 0.35 μg/mL). A similar trend was not observed in the case of lactoferrin, the value of which was insignificantly lower in people taking AR drugs (Table 1 and Table 2). Analyzing the parameters of specific immunity, it was found that the mean concentration of sIgA in resting (Me = 390.47 μg/mL) and stimulated saliva (Me = 129.04 μg/mL) was statistically significantly lower in the group undergoing AR therapy compared to the control group (US: Me = 529.03 μg/mL; SS: Me = 380.26 μg/mL) (Table 1 and Table 2).

On the other hand, the levels of IgA in the resting saliva of patients taking AR drugs were higher (Me = 374.64 μg/mL) than in the control group (Me = 223.69 μg/mL), but the difference was not statistically significant (Table 1). In the stimulated saliva, the IgA concentrations in the saliva of the study group (Me = 201.35 μg/mL) and the control group (Me = 130.33 μg/mL) were similar (Table 2). In case of neopterin, which is a non-specific indicator of cellular immune response, it was found that its concentration in both resting (Me = 3.61 nmol/L) and stimulated saliva (Me = 1.92 nmol/L) was statistically significantly lower in patients taking AR drugs compared to the control group (US: Me = 10.34 nmol/L; SS: Me = 11.55 nmol/L) (Table 1 and Table 2).

The levels of cortisol in resting (Me = 6.03 ng/mL) and stimulated saliva (Me = 5.31 ng/mL) were higher in the group receiving AR drugs than in the control group (US: Me = 2.99 ng/mL; SS: Me = 3.21 ng/mL) (Table 1 and Table 2), but this difference was statistically significant only in the case of resting saliva (Table 1).

In addition, analyzing the pH levels, amylase activity, and total protein concentrations in resting and stimulated saliva, no statistically significant differences were found between the group of patients taking AR drugs and the control group (Table 1 and Table 2). There were also no statistically significant differences in the buffering capacity of stimulated saliva between Group I and the control group (Table 3).

The occurrence of statistically significant differences between some parameters of resting and stimulated saliva was also found when comparing patients with osteoporosis who were not taking AR drugs to the control group. As in the case of people with osteoporosis taking AR drugs, compared to the control group (US: Me = 0.30 μg/mL; SS: Me = 0.35 μg/mL), significantly higher concentrations of lysozyme were found in the resting (Me = 0.72 μg/mL) and stimulated saliva (Me = 0.67 μg/mL) of patients with osteoporosis not taking AR drugs. Similarly, the mean levels of neopterin in the resting (Me = 2.17 nmol/L) and stimulated saliva (Me = 2.18 nmol/L) of patients with osteoporosis not undergoing AR therapy were significantly lower than in the control group (US: Me = 10.34 nmol/L; SS: Me = 11.55 nmol/L). Similarly, when analyzing the levels of cortisol, it was found that its levels in both resting (Me = 7.18 ng/mL) and stimulated saliva (Me = 7.36 ng/mL) of patients with osteoporosis not subjected to AR therapy were significantly higher than in the control group (US: Me = 2.99 ng/mL; SS: Me = 3.21 ng/mL). In the case of Group I, this difference was statistically significant only in resting saliva (Table 1 and Table 2).

## 4. Discussion

Saliva is the environment of the oral cavity and performs numerous functions [23,24,25,26,27]. Its composition reflects the condition of the whole organism, because apart from the ingredients produced by the salivary glands, it also contains elements diffusing from the blood [25,26]. In the case of osteoporotic patients, it was important to determine whether the use of AR therapy significantly affects the composition and properties of saliva. The obtained results were related to osteoporotic patients who were qualified for AR therapy, but in whom this therapy had not yet been implemented. Due to the importance of this problem, the saliva tests were comprehensive and concerned both resting and stimulated saliva.

The results of our own research indicate that the composition and properties of the saliva of osteoporotic patients who had been treated with AR therapy did not change significantly compared to the saliva of people with osteoporosis who had not yet received this therapy. The pH and buffering properties of saliva, the concentrations of PO_4_ and Ca ions, and the concentrations of total protein, lysozyme, lactoferrin, sIgA, IgA, neopterin, and cortisol, as well as the amylase activity, did not differ significantly between the two groups of osteoporotic patients. There was also no relationship between the duration of AR therapy in patients with osteoporosis and the examined parameters of the saliva. This was true for both resting and stimulated saliva.

However, comparing the results of the saliva tests obtained in both groups of osteoporotic patients with the control group, significant differences were found. In this comparison with the control group, the results of saliva tests in osteoporotic patients taking AR drugs are particularly noteworthy. They indicate that compared to the control group, the saliva of Group I was characterized by a significantly higher concentration of PO_4_ ions (resting saliva) and a significantly lower concentration of Ca ions (stimulated saliva), a significantly higher lysozyme concentration (resting and stimulated saliva), significantly lower concentrations of sIgA (resting and stimulated saliva) and neopterin (resting and stimulated saliva), and significantly higher concentrations of cortisol (resting saliva). No significant differences between Group I and the control group were found in terms of pH and buffering properties of saliva, total protein, lactoferrin, and IgA concentrations, or amylase activity.

Significant differences in the composition and properties of saliva were also observed between the control group and people with osteoporosis in whom AR therapy was not implemented, but they concerned a smaller number of the tested saliva parameters. Compared to the control group, it was found that the saliva of osteoporotic people not taking AR drugs was characterized by significantly higher lysozyme concentration, significantly lower neopterin concentration, and significantly higher cortisol concentration. These differences were significant in both resting and stimulated saliva.

So far, numerous papers have been published describing the levels of biomarkers associated with bone resorption and formation in various bodily fluids of patients with bone mineral changes [34,35,36,37,38,39,40]. In saliva, the following were the most frequently tested: N-telopeptide of type I collagen (NTX), C-telopeptide of type I collagen (CTX), salivary calcium, bone-specific alkaline phosphatase, and osteocalcin [37,38,39,40]. Some adverse effects of AR therapy on oral health have been highlighted recently; however, little is known about its potential side effects on the physicochemical properties of saliva. According to the analysis of the available literature, comprehensive studies of the composition and properties of saliva in patients with osteoporosis taking AR drugs, while including osteoporotic patients not using AR therapy and patients without osteoporosis, have not been conducted so far.

The saliva concentrations of Ca and PO_4_ ions presented statistically significant differences between the osteoporotic group taking AR drugs and the control group in this study. The elderly are characterized by low calcium levels associated with vitamin D deficiency, which leads to the development of secondary hyperparathyroidism [10]. Under the influence of the parathyroid hormone, the release of calcium and phosphate from bones increases via a homeostatic mechanism and by stimulating bone resorption, which is reflected in the concentration of Ca ions in the blood [41]. In our study, in the group of osteoporotic patients undergoing AR therapy, a significantly higher level of PO_4_ ions was observed in the resting saliva, along with a significantly lower level of Ca ions in the stimulated saliva, compared to the control group. The significantly lower concentration of Ca ions that we observed in stimulated saliva in the group of patients undergoing AR therapy due to osteoporosis compared to the control group may indicate the effectiveness of AR treatment, because AR drugs inhibit bone metabolism, contributing to lowering the concentration of free calcium. In this study, in the group of patients with osteoporosis who were not subjected to AR therapy, no statistically significant differences were observed in terms of Ca and PO_4_ ions. In the literature, opinions are divided on the levels of ions in the saliva of people with osteoporosis taking and not taking AR drugs. Yalçin et al. studied the effects of treatment with hormone replacement therapy (HRT), alendronate (ALN), and calcium supplements on selected parameters and the composition of saliva as well as the oral status in menopausal women with established osteoporosis [42]. Unstimulated saliva was tested, in which salivary flow rate, pH, and electrolytes (Na, K, Cl, and Ca ions) were determined before the implementation of anti-osteoporosis treatment and after three and six months from the start of the therapy. The results of the study did not confirm the effect of the implementation of anti-osteoporosis drugs on the level of Ca ions in saliva [42]. Similar results in terms of Ca ions in saliva were obtained by Buduneli et al., according to whom long-term calcium and vitamin supplementation with or without ALN also does not appear to have a significant effect on salivary Ca, K, Mg, and Na concentrations or on supragingival calculus formation [43]. On the other hand, a significant increase in Ca ions in the saliva of people with low BMD compared to people without bone mineral changes was described by Saha et al., Kumbhojkar et al., and Pereira et al. [38,39,40]. In saliva, Ca and PO_4_ ions are responsible for remineralization processes and contribute to the formation of calculus [23,24,26,27,29,30,44,45]. High levels of Ca and PO_4_ ions in saliva in the presence of poor oral hygiene are associated with an increased risk of developing periodontitis, but with a reduced incidence of caries [44,45,46]. The results of our own research indicate a significantly lower level of calcium in Group I, which suggests the possibility of impaired remineralization processes, and this may be conducive to both the development of caries and enamel erosion. On the other hand, significantly higher levels of PO_4_ ions may lead to tartar buildup; therefore, taking into account other factors, the risk of periodontal disease among this group of patients also increases. In Group II, based on the results of the concentrations of Ca and PO_4_ ions, this risk seems to be lower.

Analyzing our own research, the group of patients with osteoporosis undergoing AR therapy was distinguished from the control group by another parameter, i.e., significantly lower sIgA levels. This relationship applied to both resting and stimulated saliva. sIgA is an immunoglobulin that is specific to saliva and accounts for 60% of all antibodies present in saliva [28]. This immunoglobulin plays a role in the neutralization of bacterial toxins and enzymes, inhibits bacterial adherence, and enhances their elimination through agglutination [28,29]. Moreover, a specific immune response against *Streptococcus mutans* is provided by sIgA [28]. However, so far, the diagnostic value of sIgA as a caries biomarker has not been unequivocally proven [28]. In the studies of Miletic et al., lower sIgA levels were found in elderly people [47]. However, this relationship does not explain the results of our research, because all of the study groups—including the control group—were similar in age. Nevertheless, information about low sIgA values in the group of patients with osteoporosis taking AR drugs may indicate a potentially higher risk of developing MRONJ. According to the 2022 update of the American Association of Oral and Maxillofacial Surgeons’ Position Paper on MRONJ, the main hypotheses regarding the pathophysiology of MRONJ—apart from bone remodeling inhibition, inflammation of infection, angiogenesis inhibition, and genetic factors—now also include innate or acquired immune dysfunction [19]. Further studies of the levels of sIgA in patients with symptoms of MRONJ are recommended to confirm this hypothesis, because the level of sIgA in saliva is affected by many factors, including systemic factors such as stress [47,48].

In our study, there were statistically significant differences in lysozyme levels in Group I and Group II compared to the control group. For both groups, there was a statistically significant increase in lysozyme levels in both resting and stimulated saliva compared to the control group. Lysozyme is one of the elements of non-specific immunity. Lysozyme causes the hydrolysis of the cell walls of bacteria—especially Gram-positive bacteria [25,28]. Thanks to the biological activity of lysozyme, the formation of biofilms by cariogenic bacteria and the adhesion of *Streptococcus mutans* to hydroxyapatite are reduced [28]. According to the available literature, studies were conducted to determine whether there is a relationship between the level of lysozyme and the activity of caries [49,50]. Jentsch et al., in a study of adults followed for caries over a period of 4 years, observed that a lower caries increment was associated with lower levels of lysozyme [49]. In turn, Lertsirivorakul et al., examining the resting saliva of preschool children, noticed an increased level and activity of lysozyme in children with severe early childhood caries (S-ECC) compared to children without caries [50]. Moreover, in addition to antibacterial activity, lysozyme exhibits antiviral and antifungal activity [28]. Yeh et al., comparing the levels of lysozyme in the saliva of 595 patients, noted that the level of lysozyme derived from the parotid increases with the increase in the *Candida* load [51]. The literature also describes the relationship between the activity of lysozyme and the condition of the periodontium [52]. In their study, Surna et al. proved that the activity of lysozyme in crevicular fluid and in unstimulated saliva correlates with periodontal pocket depth in patients with gingivitis or periodontitis [52]. The results of our research indicate a significant increase in the concentration of lysozyme in the group of patients suffering from osteoporosis, regardless of taking AR drugs. This may suggest an increased susceptibility to caries and fungal infections in this patient population. The obtained results indicate the need for further research in this direction.

In both studied groups of osteoporotic patients—taking and not taking AR drugs—a statistically significant decrease in the levels of neopterin in the resting and stimulated saliva was observed in relation to the control group. Measurement of neopterin concentrations in human bodily fluids such as saliva, serum, urine, cerebrospinal fluid, and pleural fluid allows insight into a cell-mediated immune response [53,54]. Neopterin is synthesized from guanosine triphosphate (GTP) and secreted by macrophages. Macrophages are stimulated by interferon-γ, which is produced by active T lymphocytes [53,54]. Elevated levels of neopterin are noted in the course of viral diseases, autoimmune diseases, cardiovascular diseases, neurodegenerative diseases, and various cancers [53,55]. The literature also describes elevated levels of neopterin in the course of periodontitis [32,56,57]. Significantly lower levels of neopterin in both resting and stimulated saliva in both groups of patients with osteoporosis (Groups I and II) compared to controls may indicate a reduction in the cellular immune response in this population of patients, which may result in increased susceptibility to the occurrence, development, and course of various pathological processes in the oral cavity. However, further research in this direction is recommended.

In our research, a significant increase in the level of cortisol was observed in the group of patients with osteoporosis taking AR drugs in terms of resting saliva, as well as in the resting and stimulated saliva of patients with osteoporosis not taking AR drugs, compared to the control group. Cortisol belongs to the group of steroid hormones called glycosteroids. Salivary cortisol testing is often used as a marker of psychological stress and is a method for assessing the activity of the hypothalamic–pituitary–adrenal (HPA) axis [33,58,59]. Elevated levels of cortisol have important physical and psychological consequences and may trigger immunological disorders [59]. The literature describes the relationship between the administration of glucocorticoids and the decrease in BMD and muscle mass [58,60,61]. Raff et al. studied 228 elderly people, including 130 men and 98 women, of whom approximately half of the women were on HRT therapy [58]. Authors measured salivary cortisol at 7 a.m. and 11 p.m. and compared the levels of salivary cortisol results with BMD. Raff et al. found a negative correlation between salivary cortisol and BMD; however, this effect was prevented by HRT [58]. These results are consistent with the results of our own research. Osella et al. assessed the relationship between cortisol secretion, bone health, and bone loss in a cohort of women in the early postmenopausal period [62]. The study included 82 women who were not diagnosed with osteoporosis at the time of beginning of the study, and parameters of the HPA axis function were assessed, including morning and midnight salivary cortisol and BMD. The study was performed twice, one year apart. The authors claim that the HPA axis may have an impact on postmenopausal bone health, but that differences in cortisol secretion do not affect the rate of bone loss in the early postmenopausal period [62].

We are aware of the limitations of our research, which include the small size of the study groups and, in the case of patients treated with AR, the multitude of anti-osteoporosis drugs used. However, despite such a small group, statistically significant results were obtained, encouraging further research in terms of the correlation of changes in saliva with the degree of BMD loss and the condition of the oral cavity—especially in terms of the frequency of caries, enamel erosion, periodontal diseases, diseases of the oral mucosa, and analysis of the oral microbiota. In these studies, the possible impact of AR therapy should still be taken into account. In addition, due to the differences in the components of saliva related to specific and non-specific immunity, further research is recommended for the possible etiopathogenesis of MRONJ in people taking AR drugs.

## 5. Conclusions

1. The results of our research indicate that the composition and properties of the saliva of people with osteoporosis in whom AR therapy was implemented did not change significantly in comparison to the saliva of people with osteoporosis in whom this therapy had not yet been implemented. However, comparing the results of the saliva tests obtained in both groups of osteoporotic patients with the control group, significant differences were found—especially in the results of saliva tests of osteoporotic people taking AR drugs, indicating a greater number of significant changes.
2. The observed changes in the composition of saliva may be clinically significant. This particularly applies to saliva parameters related to the mechanisms of its specific and non-specific immunity, as well as parameters indicating the level of cellular immune response. These changes should be analyzed in terms of factors favoring the occurrence and development of various pathological changes within the oral cavity in osteoporotic patients. In this context, the prevention of oral infections is particularly important in the group of patients taking AR drugs, due to the risk of MRONJ. The obtained results indicate the need to conduct further comprehensive research.

## Figures and Tables

**Table 1:** Comparison of biochemical parameters in unstimulated saliva from the study groups and the control group.

Unstimulated Saliva (US)	Study Groups Patients Suffering from Osteoporosis		Control Group *(n=* 32)	*p*-Value
	With Antiresorptive Therapy (*n* = 38)	Without Antiresorptive Therapy *(n =* 16)		
pH	*n* = 38	*n* = 16	*n* = 32	0.197 ^1^
Range	5.0–8.0	5.5–7.5	5.5–8.0	
Me	6.5	7.0	7.0	
Ca (mg%)	*n* = 37	*n* = 16	*n* = 32	0.058 ^1^
Range	0.6 –14.35	4.59–17.17	3.07–15.90	
Me	5.12	5.86	5.41	
PO_4_ (mg%)	*n* = 37	*n* = 16	*n* = 32	0.020 ^1,^* ^a^ 0.027 ^2,^*
Range	5.12–71.16	10.88–44.16	5.15–66.62	
Me	23.39 ^a^	25.92	14.62 ^a^	
Total Protein (mg/mL)	*n* = 36	*n* = 16	*n* = 28	0.475 ^1^
Range	0.48–4.73	0.83–4.04	0.42–6.07	
Me	2.07	2.87	2.35	
Lactoferrin (μg/mL)	*n* = 35	*n* = 15	*n* = 28	0.755 ^1^
Range	0.36–201.00	1.10–60.10	1.48–230.00	
Me	13.20	10.83	24.23	
Lysozyme (μg/mL)	*n* = 35	*n* = 16	*n* = 28	<0.001 ^1,^*
Range	0.18–3.14	0.15–1.87	0.03–0.72	^a^ <0.001 ^2,^*
Me	0.77 ^a^	0.72 ^b^	0.30 ^a,b^	^b^ 0.004 ^2,^*
sIgA (μg/mL)	*n* = 31	*n* = 16	*n* = 29	0.009 ^1,^* ^a^ 0.011 ^2,^*
Range	39.20–1385.03	87.37–938.71	96.67–1193.54	
Me	390.47 ^a^	372.76	529.03 ^a^	
IgA (μg/mL)	*n* = 35	*n* = 16	*n* = 27	0.087 ^1^
Range	103.94–1255.88	67.48–1339.40	38.88–1125.39	
Me	374.64	331.95	223.69	
Cortisol (ng/mL)	*n* = 34	*n* = 16	*n* = 32	0.001 ^1,^*
Range	0.83–40.00	1.39–10.62	0.84–6.71	^a^ 0.033 ^2,^*
Me	6.03 ^a^	7.18 ^b^	2.99 ^a,b^	^b^ 0.001 ^2,^*
Neopterin (nmol/L)	*n* = 33	*n* = 16	*n* = 31	<0.001 ^1,^*
Range	1.14–13.27	1.22–13.28	1.35–124.30	^a^ 0.001 ^2,^*
Me	3.61 ^a^	2.17 ^b^	10.34 ^a,b^	^b^ 0.006 ^2,^*
Amylase (U/mL)	*n* = 30	*n* = 16	*n* = 29	0.254 ^1^
Range	1.68–685.00	10.06–611.10	12.15–538.57	
Me	89.64	194.00	84.99	

Legend: ^1^ Kruskal–Wallis test, * α = 0.05; ^2^ post hoc test, * α = 0.05; Me = median; indication of significant differences between the specified parameters: a-a, b-b; *n*—the number of saliva samples on which the test was performed (in some cases, the amount of saliva collected was not sufficient to perform the test).

**Table 2:** Comparison of biochemical parameters in stimulated saliva from the study groups and the control group.

Stimulated Saliva (SS)	Study Groups Patients Suffering from Osteoporosis		Control Group (*n* = 32)	*p*-Value
	With Antiresorptive Therapy *(n* = 38)	Without Antiresorptive Therapy (*n* = 16)		
pH	*n* = 38	*n* = 16	*n* = 32	0.170 ^1^
Range	5.5–8.0	6.0–8.0	5.5–8.0	
Me	7.0	7.5	7.5	
Ca (mg%)	*n* = 38	*n* = 16	*n* = 32	0.046 ^1,^* ^a^ 0.040 ^2,^*
Range	0.98–12.66	1.02–7.42	1.02–14.18	
Me	3.52 ^a^	4.08	4.51 ^a^	
PO_4_ (mg%)	*n* = 38	*n* = 16	*n* = 32	0.443 ^1^
Range	2.57–35.58	2.57–33.63	5.85–52.19	
Me	14.72	14.65	11.90	
Total Protein (mg/mL)	*n* = 36	*n* = 16	*n* = 31	0.221 ^1^
Range	0.39–4.10	0.48–3.64	0.39–6.02	
Me	1.39	2.07	1.83	
Lactoferrin (μg/mL)	*n* = 37	*n* = 16	*n* = 29	0.114 ^1^
Range	0.95–74.00	1.20–32.16	0.72–35.60	
Me	7.13	8.32	17.73	
Lysozyme (μg/mL)	*n* = 36	*n* = 16	*n* = 29	0.001 ^1,^*
Range	0.03–4.18	0.22–1.87	0.04–0.72	^a^ 0.001 ^2,^*
Me	0.64 ^a^	0.67 ^b^	0.35 ^a,b^	^b^ 0.022 ^2,^*
sIgA (μg/mL)	*n* = 35	*n* = 16	*n* = 30	0.003 ^1,^* ^a^ 0.003 ^2,^*
Range	49.60–429.03	21.63–961.29	66.60–1212.90	
Me	129.04 ^a^	168.08	380.26 ^a^	
IgA (μg/mL)	*n* = 36	*n* = 16	*n* = 30	0.410 ^1^
Range	25.14–1041.82	18.40–1076.95	22.31–1499.29	
Me	201.35	270.74	130.33	
Cortisol (ng/mL)	*n* = 37	*n* = 15	*n* = 32	0.022 ^1,^* ^a^ 0.020 ^2,^*
Range	0.77–52.00	0.75–10.62	0.75–14.20	
Me	5.31	7.36 ^a^	3.21 ^a^	
Neopterin (nmol/L)	*n* = 35	*n* = 16	*n* = 32	<0.001 ^1,^*
Range	1.15–39.56	1.19–13.05	1.46–39.23	^a^ <0.001 ^2,^*
Me	1.92 ^a^	2.18 ^b^	11.55 ^a,b^	^b^ 0.005 ^2,^*
Amylase (U/mL)	*n* = 33	*n* = 16	*n* = 30	0.723 ^1^
Range	1.68–685.00	9.04–722.00	17.01–523.09	
Me	106.80	166.99	115.00	

Legend: ^1^ Kruskal–Wallis test, * α = 0.05; ^2^ post hoc test, * α = 0.05; Me = median; indication of significant differences between the specified parameters: a-a, b-b; *n*—the number of saliva samples on which the test was performed (in some cases, the amount of saliva collected was not sufficient to perform the test).

**Table 3:** Comparison of buffer capacity of stimulated saliva from study groups and the control group.

Stimulated Saliva (SS)	Study Groups Patients Suffering from Osteoporosis		Control Group *n* = 32 (100%)	*p*-Value
	With Antiresorptive Therapy *n* = 38 (100%)	Without Antiresorptive Therapy *n* = 16 (100%)		
Low Buffer Capacity	3 (7.9%)	0 (0.0%)	2 (6.3%)	0.143 ^1^
Medium Buffer Capacity	11 (28.9%)	7 (43.8%)	4 (12.5%)	
High Buffer Capacity	24 (63.2%)	9 (56.3%)	26 (81.3%)	

Legend: ^1^ chi-squared test, α = 0.05.

## Data Availability

The data presented in this study are available upon request from the corresponding author.

## References

[1] Kanis J.A., Cooper C., Rizzoli R., Reginster J.-Y., on behalf of the Scientific Advisory Board of the European Society for Clinical and Economic Aspects of Osteoporosis (ESCEO) and the Committees of Scientific Advisors and National Societies of the International Osteoporosis Foundation (IOF) (2019). European guidance for the diagnosis and management of osteoporosis in postmenopausal women. Osteoporos. Int..

[2] Svedbom A., Hernlund E., Ivergård M., Compston J., Cooper C., Stenmark J., McCloskey E.V., Jönsson B., Kanis J.A. (2013). Osteoporosis in the European Union: A compendium of country-specific reports. Arch. Osteoporos..

[3] Hanley D.A., Adachi J.D., Bell A., Brown V. (2012). Denosumab: Mechanism of action and clinical outcomes. Int. J. Clin. Pract..

[4] Borgström F., Karlsson L., Ortsäter G., Norton N., Halbout P., Cooper C., Lorentzon M., McCloskey E.V., Harvey N.C., Javaid M.K. (2020). for the International Osteoporosis Foundation. Fragility fractures in Europe: Burden, management and opportunities. Arch. Osteoporos..

[5] Anil S., Preethanath R.S., AlMoharib H.S., Kamath K.P., Anand P.S. (2013). Impact of osteoporosis and its treatment on oral health. Am. J. Med. Sci..

[6] Devlin H., Allen P., Graham J., Jacobs R., Nicopoulou-Karayianni K., Lindh C., Marjanovic E., Adams J., Pavitt S., van der Stelt P. (2008). The role of the dental surgeon in detecting osteoporosis: The OSTEODENT study. Br. Dent. J..

[7] Streckfus C.F., Johnson R.B., Nick T., Tsao A., Tucci M. (1997). Comparison of alveolar bone loss, alveolar bone density and second metacarpal bone density, salivary and gingival crevicular fluid interleukin-6 concentrations in healthy premenopausal and postmenopausal women on estrogen therapy. J. Gerontol. A Biol. Sci. Med. Sci..

[8] Tak I.H., Shin M.H., Kweon S.S., Nam H.S., Cauley J.A., Kim O.J., Kim Y.J., Chung H.J., Kim O.S. (2014). The association between periodontal disease, tooth loss and bone mineral density in a Korean population. J. Clin. Periodontol..

[9] Kim C.S., Kim E.K., Lee K.S., Lee H.K., Choi Y.H., Hwang T.Y., Moon J.S. (2015). Relationship between bone mineral density, its associated physiological factors, and tooth loss in postmenopausal Korean women. BMC Womens Health..

[10] Bouvard B., Annweiler C., Legrand E. (2021). Osteoporosis in older adults. Joint Bone. Spine.

[11] Tella S.H., Gallagher J.C. (2014). Prevention and treatment of postmenopausal osteoporosis. J. Steroid. Biochem. Mol. Biol..

[12] Camacho P.M., Petak S.M., Binkley N., Diab D.L., Eldeiry L.S., Farooki A., Harris S.T., Hurley D.L., Kelly J., Lewiecki E.M. (2020). American association of clinical endocrinologists/ american college of endocrinology clinical practice guidelines for the diagnosis and treatment of postmenopausal osteoporosis—2020 update. Endocr. Pract..

[13] Eriksen E.F., Díez-Pérez A., Boonen S. (2014). Update on long-term treatment with bisphosphonates for postmenopausal osteoporosis: A systematic review. Bone.

[14] Fuggle N.R., Curtis B., Clynes M., Zhang J., Ward K., Javaid M.K., Harvey N.C., Dennison E., Cooper C. (2021). The treatment gap: The missed opportunities for osteoporosis therapy. Bone.

[15] Khosla S., Hofbauer L.C. (2017). Osteoporosis treatment: Recent developments and ongoing challenges. Lancet Diabetes Endocrinol..

[16] Drake M.T., Clarke B.L., Khosla S. (2008). Bisphosphonates: Mechanism of action and role in clinical practice. Mayo. Clin. Proc..

[17] Otto S., Aljohani S., Fliefel R., Ecke S., Ristow O., Burian E., Troeltzsch M., Pautke C., Ehrenfeld M. (2021). Infection as an Important factor in Medication-related Osteonecrosis of the Jaw (MRONJ). Medicina.

[18] Eastell R., Rosen C.J., Black D.M., Cheung A.M., Murad M.H., Shoback D. (2019). Pharmacological Management of Osteoporosis in Postmenopausal Women: An Endocrine Society Clinical Practice Guideline. J. Clin. Endocrinol. Metab..

[19] Ruggiero S.L., Dodson T.B., Aghaloo T., Carlson E.R., Ward B.B., Kademani D. (2022). American Association of Oral and Maxillofacial Surgeons’ Position Paper on Medication-Related Osteonecrosis of the Jaws-2022 Update. J. Oral. Maxillofac. Surg..

[20] Kendler D.L., Cosman F., Stad R.K., Ferrari S. (2022). Denosumab in the Treatment of Osteoporosis: 10 Years Later: A Narrative Review. Adv. Ther..

[21] Nisi M., Karapetsa D., Gennai S., Ramaglia L., Graziani F., Gabriele M. (2018). Conservative surgical treatment of medication related osteonecrosis of the jaw (MRONJ) lesions in patients affected by osteoporosis exposed to oral bisphosphonates: 24 months follow-up. J. Cranio-Maxillofac Surg..

[22] Kos M. (2014). Association of dental and periodontal status with bisphosphonate-related osteonecrosis of the jaws. A retrospective case controlled study. Arch. Med. Sci..

[23] Pedersen A.M.L., Sørensen C.E., Proctor G.B., Carpenter G.H., Ekström J. (2018). Salivary secretion in health and disease. J. Oral. Rehabil..

[24] Carpenter G.H. (2013). The secretion, components, and properties of saliva. Annu. Rev. Food Sci. Technol..

[25] Farnaud S.J., Kosti O., Getting S.J., Renshaw D. (2010). Saliva: Physiology and diagnostic potential in health and disease. Sci. World J..

[26] Greabu M., Battino M., Mohora M., Totan A., Didilescu A., Spinu T., Totan C., Miricescu D., Radulescu R. (2009). Saliva-a diagnostic window to the body, both in health and in disease. J. Med. Life..

[27] Humphrey S.P., Williamson R.T. (2001). A review of saliva: Normal composition, flow, and function. J. Prosthet. Dent..

[28] Wang K., Zhou X., Li W., Zhang L. (2019). Human salivary proteins and their peptidomimetics: Values of function, early diagnosis, and therapeutic potential in combating dental caries. Arch. Oral. Biol..

[29] Lynge Pedersen A.M., Belstrøm D. (2019). The role of natural salivary defences in maintaining a healthy oral microbiota. J. Dent..

[30] Hicks J., Garcia-Godoy F., Flaitz C. (2003). Biological factors in dental caries: Role of saliva and dental plaque in the dynamic process of demineralization and remineralization (part 1). J. Clin. Pediatr. Dent..

[31] Nijakowski K., Gruszczyński D., Kopała D., Surdacka A. (2022). Salivary Metabolomics for Oral Squamous Cell Carcinoma Diagnosis: A Systematic Review. Metabolites.

[32] Ozmeriç N., Baydar T., Bodur A., Engin A.B., Uraz A., Eren K., Sahin G. (2002). Level of neopterin, a marker of immune cell activation in gingival crevicular fluid, saliva, and urine in patients with aggressive periodontitis. J. Periodontol..

[33] Hellhammer D.H., Wüst S., Kudielka B.M. (2009). Salivary cortisol as a biomarker in stress research. Psychoneuroendocrinology.

[34] Greenspan S.L., Rosen H.N., Parker R.A. (2000). Early changes in serum N-telopeptide and C-telopeptide cross-linked collagen type 1 predict long-term response to alendronate therapy in elderly women. J. Clin. Endocrinol. Metab..

[35] Ravn P., Clemmesen B., Christiansen C. (1999). Biochemical markers can predict the response in bone mass during alendronate treatment in early postmenopausal women. Alendronate Osteoporosis Prevention Study Group. Bone.

[36] Garnero P. (2008). Biomarkers for osteoporosis management: Utility in diagnosis, fracture risk prediction and therapy monitoring. Mol. Diagn. Ther..

[37] Kerschan-Schindl K., Boschitsch E., Marculescu R., Gruber R., Pietschmann P. (2020). Bone turnover markers in serum but not in saliva correlate with bone mineral density. Sci. Rep..

[38] Saha M.K., Agrawal P., Saha S.G., Vishwanathan V., Pathak V., Saiprasad S.V., Dhariwal P., Dave M. (2017). Evaluation of Correlation between Salivary Calcium, Alkaline Phosphatase and Osteoporosis- A Prospective, Comparative and Observational Study. J. Clin. Diagn. Res..

[39] Kumbhojkar S.V., Kale A.D., Kumbhojkar V.R., Desai K.M. (2019). Salivary calcium as a diagnostic tool for screening of osteoporosis in postmenopausal women. J. Oral. Maxillofac Pathol..

[40] Pereira I.F., Brasileiro C.B., Kleperon N.P., Abreu M.H.N.G., Silva T.A.D., Mesquita R.A., Amaral T.M.P. (2018). Comparative study of oral and salivary parameters in patients with and without loss of bone mass. Braz. Oral. Res..

[41] Giustina A., Bilezikian J.P., Giustina A., Bilezikian J.P. (2018). Vitamin D in Clinical Medicine. Frontiers Hormal Research.

[42] Yalçin F., Gurgan S., Gurgan T. (2005). The effect of menopause, hormone replacement therapy (HRT), alendronate (ALN), and calcium supplements on saliva. J. Contemp. Dent. Pract..

[43] Buduneli N., Saygan B.H., Karaduman U., Saraç F., Karaduman M., Ayçelik N. (2008). Calcium, vitamin D supplements with or without alendronate and supragingival calculus formation in osteoporotic women: A preliminary study. Expert. Opin. Pharmacother..

[44] Fiyaz M., Ramesh A., Ramalingam K., Thomas B., Shetty S., Prakash P. (2013). Association of salivary calcium, phosphate, pH and flow rate on oral health: A study on 90 subjects. J. Indian Soc. Periodontol..

[45] Kavanagh D.A., Svehla G. (1998). Variation of salivary calcium, phosphate and buffering capacity in adolescents. Arch. Oral. Biol..

[46] Rajesh K.S., Zareena Hegde S., Arun Kumar M.S. (2015). Assessment of salivary calcium, phosphate, magnesium, pH, and flow rate in healthy subjects, periodontitis, and dental caries. Contemp. Clin. Dent..

[47] Miletic I.D., Schiffman S.S., Miletic V.D., Sattely-Miller E.A. (1996). Salivary IgA secretion rate in young and elderly persons. Physiol. Behav..

[48] Phillips A.C., Carroll D., Evans P., Bosch J.A., Clow A., Hucklebridge F., Der G. (2006). Stressful life events are associated with low secretion rates of immunoglobulin A in saliva in the middle aged and elderly. Brain. Behav. Immun..

[49] Jentsch H., Beetke E., Göcke R. (2004). Salivary analyses and caries increment over 4 years: An approach by cluster analysis. Clin. Oral. Investig..

[50] Lertsirivorakul J., Petsongkram B., Chaiyarit P., Klaynongsruang S., Pitiphat W. (2015). Salivary Lysozyme in Relation to Dental Caries among Thai Preschoolers. J. Clin. Pediatr. Dent..

[51] Yeh C.K., Dodds M.W., Zuo P., Johnson D.A. (1997). A population-based study of salivary lysozyme concentrations and candidal counts. Arch. Oral. Biol..

[52] Surna A., Kubilius R., Sakalauskiene J., Vitkauskiene A., Jonaitis J., Saferis V., Gleiznys A. (2009). Lysozyme and microbiota in relation to gingivitis and periodontitis. Med. Sci. Monit..

[53] Michalak Ł., Bulska M., Strząbała K., Szcześniak P. (2017). Neopterin as a marker of cellular immunological response. Postepy. Hig. Med. Dosw..

[54] Sucher R., Schroecksnadel K., Weiss G., Margreiter R., Fuchs D., Brandacher G. (2010). Neopterin, a prognostic marker in human malignancies. Cancer Lett..

[55] Murr C., Widner B., Wirleitner B., Fuchs D. (2002). Neopterin as a marker for immune system activation. Curr. Drug. Metab..

[56] Mahendra L., Mahendra J., Borra S.K., Nagarajan A. (2014). Estimation of salivary neopterin in chronic periodontitis. Indian J. Dent. Res..

[57] Vrecko K., Staedtler P., Mischak I., Maresch L., Reibnegger G. (1997). Periodontitis and concentrations of the cellular immune activation marker neopterin in saliva and urine. Clin. Chim. Acta.

[58] Raff H., Raff J.L., Duthie E.H., Wilson C.R., Sasse E.A., Rudman I., Mattson D. (1999). Elevated salivary cortisol in the evening in healthy elderly men and women: Correlation with bone mineral density. J. Gerontol. A Biol. Sci. Med. Sci..

[59] Montero-López E., Santos-Ruiz A., González R., Navarrete-Navarrete N., Ortego-Centeno N., Martínez-Augustín O., Rodríguez-Blázquez M., Peralta-Ramírez M.I. (2017). Analyses of hair and salivary cortisol for evaluating hypothalamic-pituitary-adrenal axis activation in patients with autoimmune disease. Stress.

[60] Compston J. (2018). Glucocorticoid-induced osteoporosis: An update. Endocrine.

[61] Di Somma C., Colao A., Pivonello R., Klain M., Faggiano A., Tripodi F.S., Merola B., Salvatore M., Lombardi G. (1998). Effectiveness of chronic treatment with alendronate in the osteoporosis of Cushing’s disease. Clin. Endocrinol..

[62] Osella G., Ventura M., Ardito A., Allasino B., Termine A., Saba L., Vitetta R., Terzolo M., Angeli A. (2012). Cortisol secretion, bone health, and bone loss: A cross-sectional and prospective study in normal non-osteoporotic women in the early postmenopausal period. Eur. J. Endocrinol..

